# Does duration of untreated psychosis predict very long term outcome of schizophrenic disorders? results of a retrospective study

**DOI:** 10.1186/1744-859X-11-21

**Published:** 2012-08-02

**Authors:** Diego Primavera, Chiara Bandecchi, Tiziana Lepori, Lucia Sanna, Eraldo Nicotra, Bernardo Carpiniello

**Affiliations:** 1 Department of Public Health, Section of Psychiatry and Psychiatric Clinic, University of Cagliari, Via Liguria 13, 09127 Cagliari, Italy; 2 Department of Psychology, University of Cagliari, Via Is Mirrionis 1, 09133 Cagliari, Italy

## Abstract

**Background:**

Studies performed to assess the relevance of duration of untreated psychosis (DUP) as a predictor of long-term outcome (i.e. follow-ups of ten years or more) are somewhat limited. The aim of this study was to evaluate the potential association between DUP and very long-term outcome (16-33 yrs) of schizophrenia by means of a retrospective design.

**Methods:**

Retrospective data obtained from clinical records were collected regarding DUP and outcome variables (number of hospitalizations; number of attempted suicides; course of illness; GAF scores at last observation) for a cohort of 80 outpatients (52 Males, 28 Females, mean age 51.0+/-11.58 years) affected by schizophrenia according to DSMIVTR attending a university community mental health centre.

**Results:**

Mean duration of follow up was 25.2 +/- 8.68 years; mean duration of untreated psychosis was 49.00 months (range 1-312 mo), with no significant difference according to gender. Patients with a shorter DUP (=/< 1 year) displayed more frequent “favourable” courses of illness (28.9% vs 8.6%) (p = 0.025), more frequent cases with limited (=/< 3) number of hospital admissions (85.7% vs 62.1%) (p = 0.047) and a better functioning (mean GAF score = 50.32+/-16.49 vs 40.26+/-9.60, p = 0.002); regression analyses confirmed that shorter DUP independently predicted a more positive outcome in terms of number of hospital admissions, course of illness, functioning (GAF scores).

**Conclusion:**

A shorter DUP appears to act as a significant predictor of better outcome in schizophrenia even in the very long-term.

## Introduction

The prognosis of schizophrenia has improved considerably since the era of Kraepelin. Nowadays, 30-50% of patients feature a fairly satisfactory outcome from a clinical and psychosocial point of view, with a consistent number achieving full recovery [1,2]. Prediction of long-term treatment-response and outcome continues to represent one of the unmet needs in schizophrenia. In recent years an increasing number of reports show that Duration of untreated psychosis (DUP), namely the time gap between the onset of psychotic symptoms and first treatment, may play a relevant role. Indeed, DUP has mostly been associated with an unfavourable outcome [3,4]. In particular, a significant association between DUP and several outcome indicators (including total symptoms, depression/anxiety, negative symptoms, positive symptoms, overall functioning and social functioning) was found at 6 and 12 months; compared to short, longer DUP was associated to a worse outcome at 6 months in several domains and patients with a long DUP were significantly less likely to achieve remission [3]. Moreover, a direct correlation has been demonstrated between longer DUP and negative prognosis, irrespective of any potential confounding factors such as gender, age at onset, age at first hospitalization, premorbid functioning, mode of onset, diagnosis, education, marital and socioeconomic status, family history of psychiatric illness, ethnicity, duration or continuity of antipsychotic treatment [3,4]. Consequently, any delay in the treatment of individuals affected by psychosis seems to constitute a highly negative prognostic factor, with early intervention being considered an important component of treatment programs. However, evidence provided with regard to the effectiveness of early interventions in improving the course and outcome of psychotic conditions is somewhat conflicting [5,6]. Moreover, the prognostic importance of DUP seems to have been demonstrated mainly in the short-middle term and in first-episode patients [3,4]. Indeed, a series of studies has shown a correlation between DUP and clinical outcome in first episode psychotic patients (FEP) [7-11]. A limited number of studies with longer follow-ups (approx 10-20 years), [12-17] seem to confirm the extension of validity of DUP as a prognostic factor even in the long term. These studies however were largely performed on patients identified at the time of or shortly prior to first treatment. Based on these premises, the aim of the present retrospective study was to evaluate the potential association between DUP and very long term outcome in a cohort of chronic outpatients affected by schizophrenia attending a community mental health centre (CMHC).

## Materials and methods

The study was approved by the Independent Ethical Commitee of Local Health Unit of Cagliari (Italy). Selection of the sample was based on a two-step process. In the first step, all patients over the age of 18 years with a diagnosis of schizophrenia with or without other comorbid psychiatric disorders according to DSMIV-TR [18] attending a university community mental health centre on 1^st^ January 2011 were identified from the centre register. In the second step, patients were selected on the basis of the following criteria: confirmed clinical diagnosis using the Structured Clinical Interview for Diagnosis of Axis I Disorders (SCID-I Research version) [19]; lack of comorbid mental retardation or organic brain disease; no discontinuity of care over at least six consecutive months since first enrolling with the centre; lastly, to enhance the retrospective evaluation of cases, clinical records were examined to ascertain their suitability for providing reliable retrospective data. All patients selected gave their written informed consent before being included in the study.All patients included in the analysis received standard care mostly provided in community mental health centers throughout Italy (psychopharmacological treatment, clinical monitoring at least on a monthly basis, home care when required, psychosocial and rehabilitation interventions tailored to patients’ needs). In line with procedures applied in a previous study [20], data were collected retrospectively from standardized clinical records routinely used in the community mental health centre, as described by the Italian version of procedures suggested by the Association for Methodology and Documentation in Psychiatry (AMDP) [21]. In particular, sociodemographic (gender, age, education, marital status, working status) and clinical data, namely age at onset of the disorder (based on first clear clinical psychopathological symptoms), age at first treatment (both pharmacological and/or psychosocial), duration of untreated psychosis (DUP) (considered ad the time interval between onset of first clear psychotic symptoms and first antipsychotic treatment), course of illness according to DSMIV-TR course specifier criteria for schizophrenia, number of inpatients admissions, number of attempted suicides, functioning according to GAF scale [22] were taken into account. Informations regarding DUP and other aspects of clinical history had been collected directly from patients’ interviews at the time of their first contact with the centre and, where available, from informants. For the purposes of the present study, DUP was considered an independent variable. Given the lack of standardized criteria available for use in distinguishing between “long” and “short” DUP, we decided to use a conservative approach dividing patients in two groups according to a mean split [4], as adopted in other studies [23]. Number of inpatient admissions, number of attempted suicides, clinical course and GAF scores were considered as dependent outcome variables. In particular, the course of illness was divided in two categories, namely “unfavourable course” (i.e “continuous” plus “episodic with intercritical residual symptoms” according to DSMIVTR) and “favourable” course (i.e “episodic without intercritical residual symptoms” plus “single episodes in partial remission” and “single episode in full remission”).

Data were entered in a data base set and evaluated by means of SPSS-18 statistical package. Student’s *t* test for unpaired data was used to evaluate differences between continuous variables. Pearson Chi square test or Fisher Exact test were used for categorical variables. All test were two-tailed ; level of statistical significance was set at p value equal to or lower than 0.05. In order to evaluate the relationship between continuous variables (GAF scores, number of hopitalizations, number of attempted suicides) as dependent variables and age at onset, age, duration of illness and duration of untreated psychosis as explanatory variables we used a multiple linear regression model. Binomial logistic regression analysis (method: enter) was used to evaluate the relationship between type of course (positive vs negative) as explained variable and sociodemographic (gender, age, marital status) and clinical variables (familiarity, substance abuse, axis I comorbidity, duration of illness, duration of untreated psychosis (short/long) as explanatory variables. Given that values of continuous variables were all positively skewed, they were log-transformed before regression analyses.

### Sample

In line with criteria illustrated previously, from an initial total of 115 patients with a diagnosis of schizophrenia identified in the fist step, 80 (52 Males, 28 Females) were ultimately included in the study. Patients considered in the study were largely middle aged, single and unemployed (28.7% of patients had a disability pension), although fairly well-educated (Table 1); no significant difference was observed between genders. The majority of subjects were affected by Paranoid Schizophrenia (n = 54, 61.4%), whilst others were affected by Disorganized Schizophrenia (n = 10, 11.4%), Catatonic Schizophrenia (n = 1, 1.1%) Undifferentiated Schizophrenia (n = 7, 11%) and Residual Type (n = 8, 9.1%). A positive family history for mental illness was revealed in 51 cases (66.7%). No differences were detected for gender distribution, mean age, education, marital status, employment status between the sample identified in the first step of the study and the final sample included in the study.

**Table 1:** Sociodemographic characteristics of the sample

	**Males (n = 52)**	**Females (n = 28)**	**Total (n = 80)**
Mean age (yrs)	47.94+/-10.29	56.57+/-12.12	51.0+/- 11.58*
Marital status (N, %)			
*Married*	4 (7.7%)	5 (17.8%)	9 (11.2%)*
*Not married*	48 (92.3%)	23 (82.2%)	71 (88.8%)
Education (mean yrs)	10.83+/-8.4	9.71+/-3.9	10.4+/-3.9*
Employment status			
*Employed*	5 (9.6%)	6 (21.4%)	11 (13.8%)*
*Unemployed*	47 (90.4%)	22 (78.6%)	69 (86.2%)

* not significant differences.

## Results

At the time of admission to the CMHC 29 patients (36.3%) were undergoing their fist episode and treatment; the remaining 51 (66.7%) had previously attended other public or private facilities. Age at onset was 24.9+/- 8.74 yrs, slightly but non significantly lower among males (males = 23.90+/-7.9; females 26.75+/-10.00, t = - 1.398, p = 0.166). 18 (22.5%) cases featured an early onset (before the age of 18 yrs). Median duration of illness was 23 yrs (range 6-68). Mean duration of follow-up was 25.0 +/- 8.7 yrs, higher, although lacking statistical significance, among females (M = 23.8+/-9.7; F = 29.1+/-15.8, t = -1.859, p = 0.067). Mean DUP was 49.0 months (range 1-312 months), without any significant gender-related differences (males = 43.4+/-72.0; females 60.9+/-91.9 months; t = -0.928, p = 0.126) and median DUP was 12 months; using the latter as splitting measure, 45 patients (56.3%) featured a “long” DUP (more than one year) and 35 (43.8%) a “short” DUP (one year or less). Distribution of patients according to DUP (“short” or “long”) was independent of gender, age at onset of illness, family history for psychiatric illness, axis I comorbidity . In the majority of patients course of the illness was “continuous” (N = 48, 60.0%), whilst in the remaining cases it was “episodic with intercritical residual symptoms” (N = 15, 18.8%), “episodic without intercritical residual symptoms” (N = 13, 16.3%), “single episode in partial remission” (n = 1, 1.1%), “single episode in full remission” (N = 1, 1.1%), or “Not otherwise specified” (N = 2, 2.4%). The course of illness was “unfavourable” in 80% (N = 63) of subjects whilst the remaining 20% (n = 15) featured a “favourable” course. A “favourable” course of illness was significantly more frequent among patients with a “short” DUP (Table 2). Moreover, a “short” DUP was significantly associated with a “low” number of hospital admissions [1-3] (85.7% of cases) compared to cases with a “long” DUP (62.1%) (p = 0.047). Additionally, mean number of hospital admissions was significantly lower among patients with a “short” DUP (1.5+/- 2.3) than among patients with a “long” DUP (3.2+/-2.5) (t = 3.675, p < 0.001) . This difference was not associated to differences in duration of illness, given that patients with a “short” DUP featured a similar duration of illness (24.1+/-12.9 yrs) to that of patients with a “long” DUP (27.7+/-1.4 yrs) (t = -1.227, p = 0.205). No difference was detected between patients with a “short” (0.16+/-0.77) and patients with a “long” DUP (0.20+/-0.53) as far as mean number of attempted suicides were concerned. Finally, patients with a “short” DUP showed a significantly higher mean GAF score (50.3+/- 16.5) than subjects with a “long” DUP (40.3+/-9.7) (t = 3.197, p = 0.002).

**Table 2:** Duration of untreated psychosis and Course of Illness

**DUP**	**Course of illness**	**Total**	
	**Favourable** **N (%)**		**Unfavourable** **N (%)**
=/< 1 year	13 (28.9)	32 (71.1)	45
> 1 year	3 (8.6)	32 (91.4)	35
Total	16 (20)	64 (80)	80

Chi square test = 3.889, df = 1, p = 0.049.

When linear regression analysis was performed, among explanatory variables duration of untreated psychosis resulted a significant predictor of two outcome variables, namely GAF score (B = - .068, SDE = .014, beta -.505, t = -4.859, sig. < .001, CI limits: -.096 / -.040) and number of hospitalizations (B = 1.491, SDE = .332, beta .467, t = 4.493, sig. < .001, CI limits: .830 / 2.152), while duration of illness predicted only number of hospitalization (B = 4.352, SDE = 2.052, beta .400, t = 2.121, sig. .037, CI limits: .264 / 8.440).

Using logistic regression analysis, among all explanatory variables, only short DUP significantly predicted poor course (β = 5.079, df = 1, p = .024).

## Discussion

DUP is generally considered a significant predictor of outcome, particularly for schizophrenic disorders [24]. Indeed, a longer DUP has been associated with a worse clinical and psychosocial outcome, frequently interpreted as a consequence of a more intense and rapid progression of the neurodegenerative process in the first years of untreated illness. This findings lends support to the importance of the early treatment of schizophrenia, repeatedly associated with a better prognosis [25-28]. However, the predictive value of DUP has been acknowledged mainly for cases at onset and with regard to the short- and medium-term periods [29], whilst its role in predicting long-term prognosis remains to be clarified [30].

In this study DUP proved to be an important predictor of clinical and functional outcome of schizophrenia even in the very long term, as attested by data showing the association between a shorter DUP and higher frequency of more favorable course of illness, reduced rates of hospitalization and better overall functioning in the cohort of chronic schizophrenic outpatients studied. As previoulsy pointed out, a limited number of studies have provided data on the impact of DUP in the long-term (i.e follow-up of 10 years or more) with very few studies reporting data regarding very long term outcome (15 yrs or more); moreover, evidence provided by these studies is somewhat contradictory. Some studies reported a significant relationship between longer DUP and worse outcome [12,16,31,32]. In particular, a study involving a sample of 58 patients with schizophrenia followed for a mean period of 15 years, demonstrated a reduction in overall functioning and a more severe psychopathology among patients with a DUP of more than 1 year compared to those with a DUP of less than 6 months [16]. On the contrary, several studies present in literature yelded opposite results [15,33-35]. In particular, a study of clinical and psychosocial outcomes in 55 adolescent patients with schizophrenia after a follow-up period of 15 years failed to demonstrate any predictive power of DUP on the different outcome measures considered [33].Moreover, in a multicenter follow-up study of 349 patients, throughout the first two years of disease DUP did not appear to be predictive of levels of social disability observed in patients fifteen years later [15]. Differences in methodology regarding the study design (i.e prospective vs retrospective), methods (i.e.standardized vs not standardized techniques for evaluating DUP), sampling (i.e first onset vs chronic or mixed cases; early onset vs not early onset) may explain the above mentioned conflictings results. However, in view therefore of the lack of published studies of both a prospective or retrospective nature on the impact of DUP in the very long-term (16 yrs or more) , the results obtained in the present survey of schizophrenic patients followed for an average of 25 years ( range 16- 33 yrs) would appear to be of particular relevance, considering that the demonstration of very long-term negative consequences of a prolonged delay in starting treatments tend to confirm the potential relevance of early treatment even on the long-term outcome of psychotic disorders [36]. However, in evaluating these results, the possibility that a longer DUP may be related to severity of the illness, and thus may be a marker than a determinant of outcome cannot be ruled out [9]. Moreover, the above mentioned lack of prospective or retrospective data in literature relating to DUP over such a long-term follow-up period implies the need for particular caution in interpreting the findings of the study. Indeed, further studies should be undertaken in an attempt to confirm the findings here reported . Likewise, caution should be applied in drawing conclusions from the findings of this study in view of its several limitations. The first limitation is represented by the retrospective nature of the data collected. This limitation is, at least in part, counterbalanced by the relatively good quality of data obtained from clinical records, which were stored by means of a structured data recording system in use in the university community mental health centre where the present study was performed. However, the lack of use of a specifically structured interview in collecting data pertaining to important aspect such as age at onset, age at first treatment, premorbid personality, and in particular DUP should be acknowledged. Similarly, the fact that only 22% of patients studied were at their first episode when first seen should be underlined. Consequently, data pertaining to onset of ilness were collected for the majority of patients after an average three years of illness, a time frame sufficient to favour recall biases both from patients and caregivers. Secondly, we acknowledge that this is a small study, with a limited number of cases examined, partly due to the selection criteria applied, including the exclusion of cases such as schizoaffective disorders, considered in other studies. This therefore considerably limits the power of the results obtained, although the inclusion in the sample only of cases for which reliable follow-up data were available probably enhanced the quality of the study. Third, the fact that the study considers a very selected sample of patients, namely only those who were in contact with the centre without interruptions for periods of six months or more should be acknowledged; patients who had died, moved away, refused to stay in treatment due to any reason, including scarce insight and/or very severe illness, or not having further need of continuing care, were not considered in out study. Therefore, what emerges from the present study should be applied only to patients who remain in long term treatment. Fourth, it is an acknowledged fact that a wide range of means of classifying “short” and “long” DUP is described in literature, (i.e. less than or exceeding 3, 6 or 12 months) and the method used in this study (DUP less than or exceeding 12 months) is not necessarily the best. Moreover, the dichotomization of DUP addresses the problem of skewness in duration of untreated psychosis, although is confounded by the loss of a great deal of information. On the other hand, the approach of dichotomizing DUP in “short” and “long” on the basis of a median split used in our study is one of the most conservative methods used applied to address these issues. Moreover, it should be pointed out that in regression analyses performed, DUP was introduced both as a continuous and a dichotomous variable, confirming in any case its value as explanatory variable predicting outcomes. Fifth, the involvement of only some potential confounding factors focusing on the relationship between DUP and outcome was excluded, including gender, familiarity for mental illness and age at onset, whilst others, such as premorbid personality, mode of onset, diagnostic subtyping could not be ruled out. However, regarding this point, it should be underlined that several recent reviews and meta-analyses reported the significance of DUP as a predictor of outcome, independent of these confounding variables [3,4].

In conclusion, the findings of future studies investigating large patient cohorts over an extended period of time will undoubtedly provide confirmation as to whether or not DUP is indeed a reliable predictor of outcome even in the very long-term, as indicated by the present study. In any case, the data presented here give further support to the hypothesis whereby early intervention, associated with an appreciable effect on the fate of patients, is capable of altering the prognosis of schizophrenia even in the long term.

## Competing interests

The authors declare that they have no competing interests.

## Authors’ contribution

Dr. DP contributed to study conception and design, analysis and interpretation of data and drafting of the manuscript; Drs. CB, TL and LS gave substantial contribution to data acquisition; Prof. EN provided for statistical analysis of data; Prof. BC contributed to the study conception and design, to the analysis and interpretation of data and to revision of the paper. All Authors have given their final approval of the text to be published.
